# Hip Osteonecrosis Is Associated with Increased Plasma IL-33 Level

**DOI:** 10.1155/2017/1732638

**Published:** 2017-01-11

**Authors:** Jinhui Ma, Wanshou Guo, Zirong Li, Bailiang Wang, Shirui Li, Peng Wang

**Affiliations:** ^1^Department of Bone and Joint Surgery, Peking University China-Japan Friendship School of Clinical Medicine, Beijing 100029, China; ^2^Department of Bone and Joint Surgery, Center for Osteonecrosis and Joint Preserving & Reconstruction, China-Japan Friendship Hospital, Beijing 100029, China; ^3^Department of Endocrinology, China-Japan Friendship Hospital, Beijing 100029, China

## Abstract

The recently discovered IL-33 as an IL-1 cytokine family member has been proved to be specifically released from osteonecrotic bones. We aimed to investigate the potential role of IL-33 in the development of osteonecrosis of femoral head (ONFH). Forty patients diagnosed with ONFH and forty age-, sex-, and body mass index- (BMI-) matched healthy subjects were included in this prospective study between March 2016 and September 2016. A commercially available ELISA kit was used to test the level of plasma IL-33. The IL-33 levels were compared among different ARCO stages, CJFH types, and etiology groups. Plasma IL-33 levels were significantly higher in the ONFH patients than that in the control subjects. The levels of IL-33 did not differ significantly among the ONFH patients with different ARCO stages. The IL-33 levels of patients with CJFH type L3 were significantly higher than that of patients with types L1 and L2. No significant differences were observed in IL-33 levels between steroid-induced, alcohol-induced, and idiopathic patients. Our findings seem to indicate that IL-33 effects may be detrimental during ONFH, which appeared to be associated with the prognosis of ONFH. The IL-33 deserves particular attention in the pathogenesis of ONFH.

## 1. Introduction

Nontraumatic osteonecrosis of the femoral head (ONFH), also known as avascular necrosis, is a refractory and progressive disease that commonly affects young patients and has a poorly understood etiology and pathogenesis [1]. Without effective treatment, ONFH can progress and might eventually lead to femoral head collapse and degenerative changes to the hip joint [2, 3]. Nontraumatic ONFH has been associated with corticosteroid usage, chronic alcohol consumption, infection, hyperbaric events, storage disorders, marrow infiltrating diseases, coagulation defects, immoderately low or high temperatures, and some autoimmune diseases [4, 5].

The pathogenesis of nontraumatic ONFH is not completely clear, but it may be attributed to vascular injury, altered lipid metabolism/fat emboli, cell and bone death, mechanical stress, disruptive immune system, and defective bone repair [4, 6]. Previous study has shown that femoral head osteonecrosis may be caused by disruption of the immune system via lipopolysaccharide- (LPS-) activated toll-like receptor 4 (TLR4) signalling [6]. In addition, some defective repair processes including delayed new bone formation, excessive bone resorption, and tissue fibrosis instead of new bone formation have been observed after ONFH, which are suggestive of pathologic bone remodeling during the repair of the necrotic bone [7, 8]. Meanwhile, it has been shown that the immune system is substantially involved in the regulation of bone homeostasis and that chronic inflammation in particular can disturb this balance [9]. Therefore, abnormal immune responses may contribute to the pathogenesis of ONFH by impacting bone remodeling.

Cytokines are a large group of proteins, peptides, and glycoproteins secreted by cells, which could regulate immune responses and immune response cell function by combining the corresponding receptors [10]. Cytokines with diverse biological functions including growth, differentiation, and activation are also known to play an important role in regulating the balance between osteoclasts and osteoblasts and determining the rate of bone remodeling [10]. IL-33, the most recently discovered member of the IL-1 family of cytokines, was initially recognized as an orphan receptor associated with inflammatory and autoimmune diseases [11]. Early studies revealed that it is expressed in intestine of patients with Crohn's disease, blood vessels of inflamed tonsils, and synovium of patients with RA and that this expression appeared to be associated with the severity of the inflammatory condition [12]. And IL-33 is now believed to be constitutively expressed in human bone and particularly released by cells undergoing necrosis from the osteoblasts, adipocytes, and osteocytes rather than through active secretion [13–16]. Recent studies suggest that IL-33 may act as a proinflammatory cytokine in asthma, septic shock, fibroproliferative diseases, collagen vascular diseases, pleural malignancy, and cardiovascular diseases [17]. During ONFH, IL-33 may also play a role by impacting bone remodeling both directly and indirectly after it is released from osteonecrotic bones [18]. However, it is still unknown whether IL-33 increases with the repair of necrotic bone or whether IL-33 acts as a positive or a negative effect after ONFH.

The purpose of this study was to investigate the plasma level of IL-33 in the patients with ONFH. We hypothesized that after ONFH, necrotic bone stimulates the inflammatory cytokine, IL-33, and expression and that IL-33 is an important player in the development and prognosis of ONFH. An evaluation of the role of IL-33 would reveal potential mechanisms responsible for disruptive immune responses after ONFH, which might provide a novel nonsurgical therapeutic approach for ONFH.

## 2. Materials and Methods

### 2.1. Study Population

This was a prospective clinical control study. Forty patients (40 consecutive hips) diagnosed with unilateral nontraumatic osteonecrosis of femoral head (ONFH) between March 2016 and September 2016 were included in this study. The patients were diagnosed with ONFH based on clinical history, physical examination, and radiological evaluations (X-ray and MRI) by orthopedic surgeons in our department. The inclusion criterion was a diagnosis of unilateral nontraumatic ONFH. Patients presenting the following criteria were excluded: history of trauma, active infection of the affected hip, inflammatory diseases, cardiovascular diseases, immunodeficiency, HIV infection, diabetes mellitus, renal disease, or previous surgery on the hip with ONFH. Forty age-, sex-, and body mass index- (BMI-) matched healthy subjects were simultaneously recruited as the controls. The study was approved by China-Japan Friendship Hospital (CJFH), and the methods were performed in accordance with the Declaration of Helsinki. Written informed consent was obtained from all subjects or their guardians.

**Figure 1:**
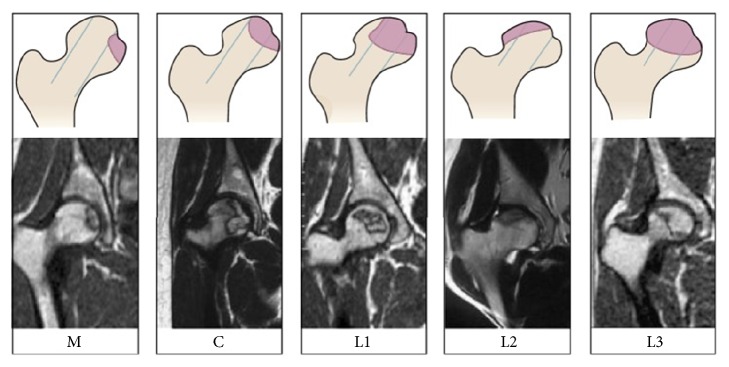
Schematic diagram and magnetic resonance image of China-Japan Friendship Hospital (CJFH) classification for osteonecrosis of the femoral head based on three pillars [19]. Type M: necrosis involves the medial pillar. Type C: necrosis involves the medial and central pillars. Type L1: necrosis involves the three pillars but the partial lateral pillar was preserved. Type L2: necrosis involves the entire lateral pillar and part of the central pillar. Type L3: necrosis involves the three pillars including the cortical bone and marrow.

The included patients were evaluated both clinically and radiologically using the Harris hip score (HHS) [19], CJFH type (Figure 1) [20], and the ARCO classification system [21]. The characteristics of the included ONFH patients are presented in Table 1. According to ARCO stage, 4 patients had stage II disease, 20 patients had stage III disease, and 16 patients had stage IV disease. CJFH types were as follows: L1, 12 patients; L2, 18 patients; L3, 10 patients; M, 0 patients; and C, 0 patients. The mean patients' age was 49.2 ± 12.4 years (range, 18–69 years), of which 31 were male and 9 were female. The average body mass index (BMI) was 24.2 ± 3.2 kg/m^2^ (range, 18.7–31.2 kg/m^2^). The mean HHS for all patients was 62.6 ± 14.6 score (range, 38–95 score). ONFH was idiopathic in 11 patients, secondary to steroid use in 14 patients, and associated with alcohol use in 15 patients. The patients were divided into two groups according to whether the lateral pillar of the femoral head (LPFH) was preserved [22]: LPFH and non-LPFH groups. The LPFH group consisted of 8 patients with the preservation of the lateral pillar of the femoral head (including CJFH type L1). The non-LPFH group consisted of 32 patients without the preservation of the lateral pillar of the femoral head (including CJFH type L2 and L3). Also, the patients were grouped into two categories according to whether the femoral head collapsed or not. The precollapse group consisted of 4 patients with stage ARCO II, and the postcollapse group consisted of 36 patients with stage ARCO III and IV. After dividing the patients into the different groups, the IL-33 levels were compared within each of the groups.

### 2.2. Staging and Typing

**Figure 2:**
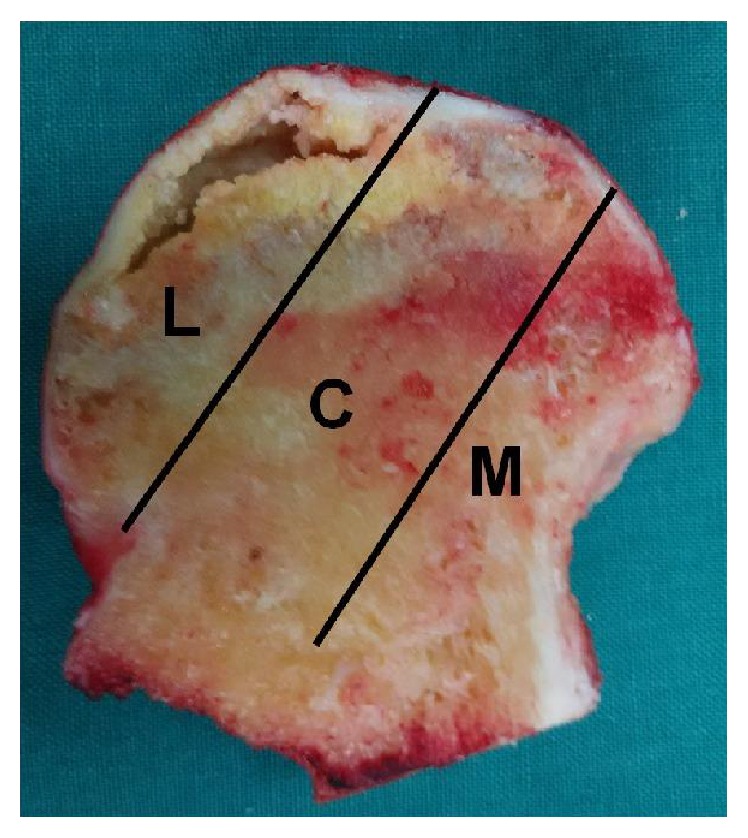
Image of coronal section of the femoral head showing three pillars of the femoral head: lateral (30%), central (40%), and medial (30%) [21].

The stages by ARCO classification system were stage II in 4 patients, stage III 20 patients, and stage IV in 16 patients. All subjects with ONFH underwent an MRI or CT evaluation according to CJFH type [20] (Figure 1) based on three pillars (Figure 2) [23]. According to the involvement of necrosis in the three pillars on a mid-coronal section on MRI or CT, ONFH location was divided into three types (M, C, and L), and the intact degree of the lateral pillar was divided into subtypes (L1, L2, and L3). Using this type to predict the prognosis of the patients with ONFH and the efficacy of joint-preserving surgery for ONFH [20], the CJFH types were type L1 in 12 hips, type L2 in 18 hips, and type L3 in 10 hips.

### 2.3. IL-33 Measurements

Blood samples were collected in sterile anticoagulation tubes from the included patients and the healthy controls, and the samples were centrifuged at 1600 rpm × 6 min to obtain plasma. The plasma was immediately frozen and stored at −80°C for analysis later. The plasma samples were tested for IL-33 levels using a commercially available ELISA kits (RayBiotech. Inc., Atlanta, USA) by following the user manual. The sensitivity of the kit is less than 2 pg/mL (range 2–500 pg/mL). The IL-33 levels were measured by two experienced independent investigators using the same instrumentation who were unaware of the study design in order to enhance measurement accuracy. All samples were duplicated during measurements. The two investigators had the same professional qualifications and were trained before the initial measurement. The data extraction and quality assessment were independently performed by two of the authors (WS. G and BL. W). If there were any disagreements, all of the authors discussed until consensus can be reached.

### 2.4. Statistical Analysis

The data were analyzed using SPSS version 19.0 statistical software (SPSS Inc., Chicago, IL, USA). Quantitative variables are reported as mean ± standard deviation (SD). Nonpaired *t*-tests were used to compare the IL-33 levels between different groups. One-way analysis of variance was used to compare IL-33 levels among different stage, type, and etiology groups. For statistically significant differences, groups were compared using the least significant difference (LSD) test. Pearson's correlation test was used to identify the correlation between the IL-33 levels and length of disease history. All tests were two-tailed at the 5% level of significance.

## 3. Results

Demographic data were comparable between the two groups (Table 2). Plasma IL-33 levels were significantly higher in the patients with ONFH (11.48 ± 8.34 pg/mL) than that in the control subjects (5.30 ± 4.36 pg/mL) (*P* < 0.001). The mean plasma IL-33 levels among the ONFH patients in the different etiologies, ARCO stages, and CJFH types are shown in Table 3. Despite the different ONFH etiologies (steroid use, excessive alcohol intake, or idiopathic origin), there were no significant differences between the cases with respect to the IL-33 levels (*P* = 0.260).

The levels of plasma IL-33 were 7.54 ± 5.13 pg/mL, 14.11 ± 10.70 pg/mL, and 9.19 ± 3.38 pg/mL in the ONFH patients with ARCO stage II, stage III, and stage IV, respectively. The levels of IL-33 did not differ significantly among the ONFH patients with different ARCO stages (*P* = 0.129). Although the postcollapse group showed a tendency of higher IL-33 level than the precollapse group, no significant differences were observed between the two groups (*P* = 0.324).

**Figure 3:**
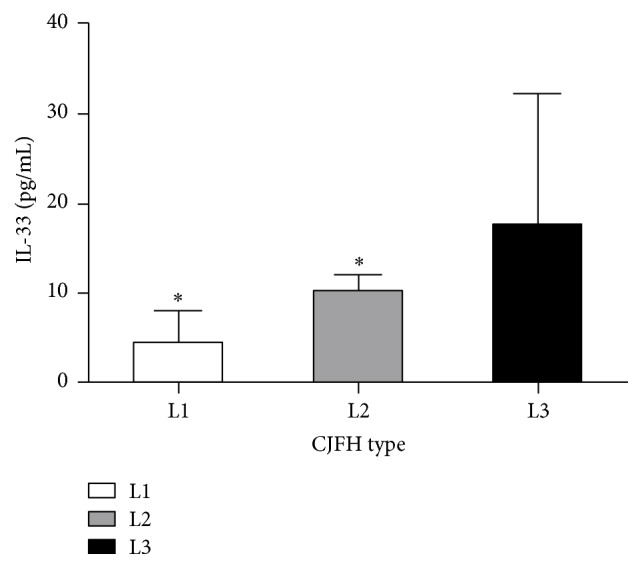
The IL-33 levels of CJFH type L3 patients were significantly higher than that of CJFH types L1 (*P* = 0.003) and L2 (*P* = 0.028) patients. ^*∗*^*P* < 0.05 compared with patients with type L3.

**Figure 4:**
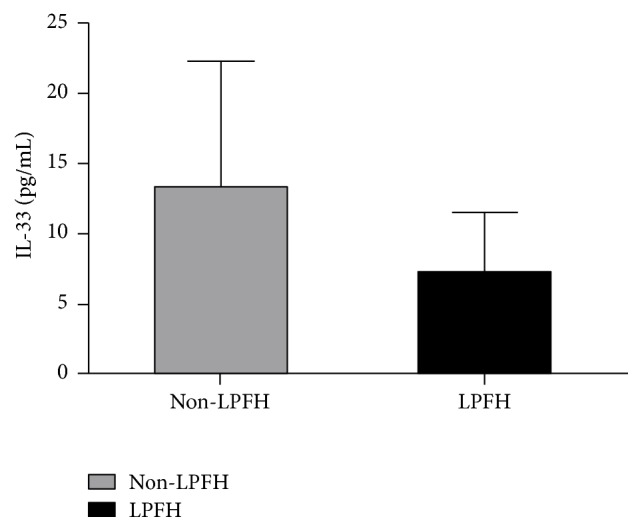
The IL-33 levels were significantly higher in the non-LPFH group than that in the LPFH group (*P* = 0.034).

The levels of plasma IL-33 were 7.27 ± 4.16 pg/mL, 10.86 ± 0.86 pg/mL, and 17.67 ± 14.55 pg/mL in the ONFH patients with CJFH types L1, L2, and L3, respectively. The IL-33 levels of CJFH type L3 patients were significantly higher than that of CJFH types L1 (*P* = 0.003) and L2 (*P* = 0.028) patients, while the IL-33 levels of patients with CJFH type L2 were not significantly different with that of patients with CJFH type L1 (*P* = 0.210) (Figure 3). Also, the IL-33 levels were significantly higher in the non-LPFH group than that in the LPFH group (*P* = 0.034) (Figure 4).

The results of Pearson's correlation test between the IL-33 levels and length of disease history for the ONFH patients showed that the IL-33 level (11.48 ± 8.34 pg/mL) did not significantly correlate with the length of disease history (38.9 ± 54.1 months) (*r* = −0.136, *P* = 0.403).

## 4. Discussion

In the present study, our findings indicated that plasma IL-33 levels were significantly higher in the ONFH patients than that in the control subjects. And the IL-33 levels of patients with CJFH type L3 were significantly higher than that of patients with types L1 and L2. However, the levels of IL-33 did not differ significantly among the ONFH patients with different ARCO stages. No significant differences were observed in IL-33 levels between steroid-induced, alcohol-induced, and idiopathic patients.

The balance between osteoclasts and osteoblasts which determines the rate of bone remodeling could be disturbed by several molecular pathways. A variety of studies in vitro and in vivo have been conducted to investigate the effect of cytokines on osteoblasts and osteoclasts and their ultimate effect on bone resorption and formation [10, 24]. IL-33, as a proinflammatory cytokine, has been proved to be specifically released from osteonecrotic bones [18]. And our results showed that the elevated IL-33 levels that were observed in the patients with ONFH also suggested this finding. During ONFH, IL-33 could modulate bone remodeling in both direct and indirect way after it is released from osteonecrotic bones [18]. IL-33 exerts its direct effects on osteoclastogenesis through the toll-like/IL-1 receptor ST2 [11]. The stimulatory effect of IL-33 on osteoclastogenesis may be in part direct. And Mun et al. [25] pointed that IL-33 stimulated formation of functional osteoclasts from human CD14(+) monocytes, enhanced expression of osteoclast differentiation factors including TNF-*α* receptor-associated factor 6 (TRAF6), nuclear factor of activated T cells cytoplasmic 1, c-Fos, c-Src, cathepsin K, and calcitonin receptor and eventually induced bone resorption. The property of IL-33 that regulates the inflammatory response and vascularization through modulating the recruitment and behavior of inflammatory cells may explain the indirect effect on bone remodeling during ONFH [18]. In addition, IL-33 may stimulate osteoclasts differentiation and activation and disturb necrotic bone repair by elevating vascular permeability [25, 26]. As a result, the normal balance of bone metabolism was disturbed and osteonecrosis of femoral head would progress.

Our study showed that the ONFH patients with CJFH type L3 hips had a higher IL-33 level than those with type L1 or L2. Also, the IL-33 levels were significantly higher in the patients without LPFH than that with LPFH. Previous studies [20, 22] in our institution have proven that the ONFH patients with CJFH type L3 hips involving all the three pillars of the femoral head had poor prognosis. Furthermore, the efficacy of joint-preserving surgery for patients with ONFH involving the lateral pillar was unsatisfactory [22]. In the patients with ONFH undergoing bone grafting through a window at the femoral head-neck junction, those with necrotic lesions involving the lateral pillar showed high surgical failure rates [27]. This indirectly illustrated that the increased IL-33 appeared to be related to the prognosis of ONFH and might act as a predictive factor for the efficacy of joint-preserving surgery in the treatment of ONFH.

Excessive corticosteroid administration is thought to be the primary risk factor in nontraumatic osteonecrosis [28]. Until now, the underlying molecular mechanisms of steroid-induced ONFH are still unknown [24]. Some researchers thought that the pathogenesis of corticosteroid-induced osteonecrosis may be due to reduced blood flow by numerous mechanisms, including marrow adipocytic hypertrophy leading to sinusoidal compression, venous stasis and, eventually, obstruction of the arteries and arterial occlusion by fat emboli and lipid-loaded fibrin-platelet thrombi [5]. Previous studies have shown that inflammatory cytokines are increased during the development of steroid-induced osteonecrosis, such as IL-1, IL-2, IL-4, IL-6, IL-10, GM-CSF, IFN-*γ*, and TNF-*α* [10]. Interestingly, we did not find that the increased plasma IL-33 level was related to risk factors of ONFH in our study, and there were no significant differences between steroid-induced, alcohol-induced, and idiopathic patients. Corticosteroid-induced osteonecrosis, which possesses the characteristic of decreasing bone formation, promoting osteoclastic resorption, impairing bone cell survival, and strengthening adipocytic differentiation, may be largely attributed to osteoblast and osteocyte apoptosis, rather than necrosis [24, 29, 30]. It is extremely important to note that IL-33 is not released during apoptosis. On the contrary, IL-33 is mainly retained inside apoptotic bodies during apoptosis, therefore preventing its accidental release [18]. In theory, the level of IL-33 should be decreased in steroid-induced ONFH. And low IL-33 levels in the patients with steroid-induced ONFH were observed in the present study.

The present study has some limitations. Firstly, the sample size is relatively small, and the analysis was not stratified for age and sex. Secondly, our observations were limited to inpatients, with no long-term follow-up. This marker may reflect the progression profile of osteonecrosis more precisely if it is based on the results from larger and more long-term clinical observations. Thirdly, the sensitivity and specificity of plasma IL-33 level have not been assessed. Fourth, we did not analyze the relationship between the necrotic area and the levels of plasma IL-33 in ONFH. Fifth, the controls were only matched in age, sex, and BMI, but not in other factors, such as alcohol intake. Last, the ONFH patients with stage ARCO I were not included in the study because most of them would not seek treatment in the absence of symptoms. Moreover, further studies are needed to clarify the effects of IL-33 on the natural progression outcomes and the joint-preserving surgery outcomes of patients with ONFH.

In conclusion, our findings seem to indicate that IL-33 effects may be detrimental during ONFH, which appeared to be associated with the prognosis of ONFH. The application of cytokines in the prognosis and therapy of ONFH shows promise; our findings may promote the development of novel therapies for the treatment of ONFH targeting IL-33. However, further experimental and clinical studies are still needed to investigate the mechanisms of IL-33 involved in the pathogenesis of ONFH and to determine the therapeutic potential of IL-33 blockade to improve bone repair after ONFH.

## Figures and Tables

**Table 1:** The demographics of patients with ONFH.

Demographic	Number/mean (SD)
Patients (M/F)	40
Male	31
Female	9
Mean age, year	49.2 (12.4)
Mean BMI, kg/m^2^	24.2 (3.2)
Mean HHS, score	62.6 (14.6)
Mean length of disease history, month	38.9 (54.1)
Etiology	
Idiopathic	11
Corticosteroids	14
Alcohol	15
ARCO stage	
Stage II	4
Stage III	20
Stage IV	16
CJFH classification	
L1	12
L2	18
L3	10

**Table 2:** Demographic data of ONFH group and control group.

	ONFH group (*n* = 40)	Control group (*n* = 40)	*P* value
Age (years)	49.2 ± 12.4	49.6 ± 16.0	0.895
Gender (male/female)	31/9	30/10	0.793
Height (cm)	168.3 ± 6.8	165.3 ± 9.0	0.099
Weight (kg)	69.0 ± 12.4	65.7 ± 9.7	0.192
BMI (kg/m^2^)	24.2 ± 3.2	24.0 ± 2.7	0.743

**Table 3:** Plasma IL-33 levels between different groups.

	Group	IL-33 level (pg/mL)	*P* value
Etiology	Corticosteroids	9.07 ± 3.27	*P* = 0.260
	Alcohol	14.14 ± 11.17	
	Idiopathic	10.94 ± 7.96	

ARCO stage	II	7.54 ± 5.13	*P* = 0.129
	III	14.11 ± 10.70	
	IV	9.19 ± 3.38	

Collapse	Precollapse	7.54 ± 5.13	*P* = 0.324
	Postcollapse	11.92 ± 8.56	

CJFH type	L1	7.27 ± 4.16	*P* = 0.010
	L2	10.86 ± 0.86	
	L3	17.67 ± 14.55	

Lateral pillar	LPFH	7.27 ± 4.16	*P* = 0.034
	Non-LPFH	13.29 ± 9.06	
